# Interaction of milk proteins and Binder of Sperm (BSP) proteins from boar, stallion and ram semen

**DOI:** 10.1186/s12958-015-0093-1

**Published:** 2015-08-15

**Authors:** Geneviève Plante, Marie-France Lusignan, Michel Lafleur, Puttaswamy Manjunath

**Affiliations:** Department of Medicine, Université de Montréal, C.P. 6128, Succ. Centre Ville, Montréal, Québec Canada H3C 3J7; Maisonneuve-Rosemont Hospital Research Center, 5415 L’Assomption blvd, Montréal, Québec Canada H1T 2M4; Department of Chemistry, Center for Self-Assembled Chemical Structures, Université de Montréal, C.P. 6128, Succ. Centre Ville, Montréal, Québec Canada H3C 3J7; Puttaswamy Manjunath, Maisonneuve-Rosemont Hospital Research Center, 5415 L’Assomption blvd, Montréal, Québec Canada H1T 2M4

**Keywords:** Sperm preservation, Milk extender, Caseins, Whey proteins, Protein-protein interaction, BSP proteins

## Abstract

**Background:**

Mammalian semen contains a family of closely related proteins known as Binder of SPerm (BSP proteins) that are added to sperm at ejaculation. BSP proteins extract lipids from the sperm membrane thereby extensively modifying its composition. These changes can ultimately be detrimental to sperm storage. We have demonstrated that bovine BSP proteins interact with major milk proteins and proposed that this interaction could be the basis of sperm protection by milk extenders. In the present study, we investigated if homologous BSP proteins present in boar, stallion and ram seminal plasma display a similar affinity for the milk proteins in order to assess whether the mechanism of sperm protection by milk for these species could be general.

**Methods:**

Skim milk was incubated with seminal plasma proteins (boar, stallion and ram), chromatographed on a Sepharose CL-4B column and protein fractions were analyzed by immunoblotting.

**Results:**

Boar, stallion and ram BSP proteins displayed affinity for a milk protein fraction (F1) mainly composed of α-lactalbumin, β-lactoglobulin, and κ-casein. They also had affinity for another milk protein fraction (F2) composed mostly of casein micelles. However, stallion BSP showed higher affinity for the fraction (F1).

**Conclusions:**

These results further extend our view that the association of BSP proteins with milk proteins could be a general feature of the mechanism of mammalian sperm protection by milk to prevent detrimental effect of prolonged exposure of sperm to seminal plasma.

## Background

Egg yolk (EY), heated skim milk (SM) and whole milk are components commonly used in extenders for sperm preservation (reviewed in [1, 2]). Being products of animal origin, their compositions are not constant, and moreover they present potential risks of microbial contamination of semen. Because of these drawbacks, there is a keen interest to find substitutes. The development of novel extenders free of products of animal origin is difficult considering that the mechanisms by which EY and milk protect sperm are poorly understood.

Bovine seminal plasma contains a family of proteins designated as Binder of SPerm (BSP) proteins, which have been extensively characterized [3–6]. These proteins positively modulate the induction of sperm capacitation, a process that is deemed to be essential for fertilization [7, 8]. However, in the context of sperm storage, BSP proteins are detrimental to sperm as they extract cholesterol and phospholipids from sperm membranes (reviewed in [2, 9]). We previously demonstrated that the low-density lipoproteins (LDL) of EY interact with BSP proteins and that this interaction prevents cholesterol and phospholipid extraction from the sperm membrane, thereby protecting sperm during preservation (reviewed in [2]).

Whole milk and SM used in extenders are also known to protect sperm during storage. While whole milk contains lipoproteins, which could bind BSP proteins and protect sperm, SM does not, and yet is as efficient as whole milk in protecting sperm [10–12]. Based on those observations, we postulated that the milk proteins could be involved in sperm protection. We have shown that casein micelles isolated from milk could interact with BSP proteins, the detrimental factors to sperm membranes [13]. The association of casein micelles with BSP proteins was shown to preclude cholesterol and phospholipid extraction from membranes induced by BSP proteins, while maintaining sperm viability and motility during sperm storage [13]. Further studies showed that bovine BSP proteins bind to several milk proteins, namely casein micelles, α-lactalbumin and β-lactoglobulin [14]. These studies led us to propose that the interaction between milk proteins and bovine BSP proteins is the basis for sperm protection during storage using milk-based extenders.

Bovine species express three BSP members: BSP1, BSP3 and BSP5 [3, 4]. Previous results showed that BSP genes and proteins are in fact a superfamily [5, 6]. Homologs of BSP proteins have been isolated and characterized from the seminal plasma (SP) or seminal vesicle secretions of many mammals, including bison [15], goat [16], stallion [17, 18], boar [8, 19] and ram [20]. In addition, a BSP1-like proteins has recently been detected in buffalo, camel and alpaca [21, 22].

Interestingly, milk extender is used for conservation of semen from stallion (reviewed in [23]), goat (reviewed in [24]), ram (reviewed in [12]) and buffalo (reviewed in [25]). Phosphocaseinates, a milk component, has also been used to preserve stallion semen [26–28]. More recently, an extender containing whey proteins has been used to preserve boar semen [29]. BSP homologs have been identified in the semen of all these species. Therefore, we postulated that the mechanism underlying sperm protection by milk in bovine species could include similar features for all those mammals. It should be noted that many differences exist between semen from different mammalian species including seminal plasma composition, and protein concentration. These factors could have an impact on semen conservation and therefore it is essential to determine the general features as well as the putative specific characteristics of BSP proteins—milk fractions interactions for each species in order to develop a detailed view of the mechanism of sperm preservation.

The goal of the current study was to determine whether BSP homologous proteins found in boar, ram and stallion seminal plasma have an affinity for milk proteins similar to the BSP proteins in bovine species. Thus, we investigated by gel filtration and immunoblot the affinity of BSP homologous proteins found in the SP of these species for the milk proteins and compared the results with those obtained with BSPs from bovine species. Commercial milk-based extenders generally include glycerin and antibiotics [1], but we focused our work on BSP protein interactions with milk proteins to highlight their specific contribution in sperm preservation. Based on these results, we believe that the mechanism of sperm protection by milk proteins previously described for BSP from bull could be broadened to include BSP proteins from more species of farm animals.

## Methods

### Materials

Tris(hydroxymethyl)aminomethane (Tris) was purchased from Sigma (St-Louis, MO) and Sepharose CL-4B and Sephadex G-25 medium were from Pharmacia Biotech Inc (Baie d’Urfé, QC, Canada). Acrylamide and bisacrylamide were purchased from MP Biomedical (Irvine, CA). Sodium-dodecyl sulfate (SDS) and other electrophoresis products were from Bio-Rad (Mississauga, ON, Canada). Low molecular weight (LMW) calibration kit was from GE Healthcare (Baie d’Urfé, QC, Canada). Immobilon-P polyvinylidene fluoride (PVDF) membranes were purchased from Millipore (Nepean, ON, Canada). Western Lightning Chemiluminescence Reagent kit was from Perkin-Elmer Life Sciences (Boston, MA). All other chemicals used were of analytical grade and obtained from commercial suppliers.

### Preparation of skimmed milk and milk fractions

Skimmed milk was purchased at a local food store. It generally contains less than 0.1 % fat (mostly triglycerides). Before use, the skimmed milk was heated in a water bath maintained at 92–95 °C for 10 min. The heated skimmed milk was then cooled at room temperature and filtered through metal mesh to remove the coagulum [30]. The skimmed milk fraction 1 (milk F1) and fraction 2 (milk F2) were prepared by gel filtration on Sepharose CL-4B column as described previously [14].

### Preparation of seminal plasma (SP) proteins

Semen used in this study was collected from fertile animals that were handled by qualified technicians, according to the Guide for the Care and Use of Agricultural Animals established by the Quebec Ministry of Agriculture and Fisheries. Stallion semen (pool of 3 ejaculates) was obtained from the Veterinary Medical School (St-Hyacinthe, Qc, Canada). Ram semen (a pool of over 100 ejaculates) was provided by the Centre d’Insémination Ovine du Québec (LaPocatière, Qc, Canada). Alcohol precipitates of SP proteins from stallion and ram semen were prepared as described previously [3, 18, 20]. Boar semen (pool of 3 ejaculates) was obtained from F. Ménard Inc. (St-Pie-de-Bagotte, Qc, Canada). Boar SP proteins were prepared as previously described [8] and concentrated using an YM-3 membrane (molecular weight cut-off, 3000 Dalton) in Amicon stirring ultrafiltration cell.

### Isolation of BSP proteins from boar, stallion and ram SP proteins

Gelatin-adsorbed ram and stallion BSP proteins were isolated as described previously [20]. Briefly, gelatin purified from calf skin was coupled to Affi-gel 15 (Bio-Rad) as previously described [31]. Lyophilysed seminal plasma proteins (~130 mg) were then dissolved in 0.05 M phosphate-buffered saline (PBS) and loaded on a gelatin–agarose column (1.5 **×** 28 cm) at a flow rate of 30 ml/h. The adsorbed proteins were eluted with PBS containing 7 M urea, pooled, concentrated to ~3 ml and desalted at room temperature on a Sephadex G-25 column (1.5 × 25 cm) equilibrated with a 50 mM ammonium bicarbonate solution. The eluted proteins were then freeze-dried and stored at 4 °C. Purified boar BSP1 was prepared by a combination of chondroitin sulfate B-affinity chromatography and reverse-phase-high performance liquid chromatography as previously described [8].

### Generation of polyclonal antibodies directed against stallion and ram BSP proteins

Polyclonal antibodies were raised in rabbits by the Biotechnology Research Institute (Montréal, QC, Canada) using gelatin-adsorbed ram or stallion proteins dissolved in saline (1 mg/ml). Polyclonal antibodies were purified from anti-sera by affinity chromatography on a protein A-Sepharose column. Their specificity was assessed by immunoblot analysis as described previously [32].

### Gel filtration chromatography

SP proteins and gelatin-bound ram proteins were dissolved in Tris-buffered saline (20 mM Tris–HCl, 150 mM NaCl, 0.02 % sodium azide, pH 7.4; TBS). Skim milk, milk F1 or milk F2 prepared as described above were filtered through a 5-μm filter. SP proteins (boar, ram and stallion), purified boar BSP1 or gelatin-bound ram BSP proteins were filtered through a 0.45-μm filter. Mixtures of SP (BSP proteins) with milk, milk F1 or milk F2 were incubated 1 h at room temperature before loading on a Sepharose CL-4B column.

Gel filtration chromatography was carried out on a Sepharose CL-4B column (78 × 2.5 cm) equilibrated with TBS at room temperature at a flow rate of 70 ml/h. Fractions of 3.2 ml were collected and their absorbance was measured at 280 nm. The presence of BSP proteins in the various fractions was analyzed by immunoblotting using respective polyclonal antibodies.

The concentration of BSP protein homologs in the SP varies from species to species [2]. In bovine species, the BSP proteins constitute the major portion of the total SP proteins (~50 %). Approximately 20–30 % of the total proteins in stallion and ram SP correspond to the BSP proteins whereas in boar, the amount of BSP1 is ~1 % of the total SP proteins. Bovine semen is generally diluted with milk extender at a dilution ratio of 1:10 to as high as 1:50 depending on the sperm count. In our previous studies with bovine species, we used 1:20 dilution ratio (corresponds to 1.75 mg SP proteins and 35 mg milk proteins) to study the interaction of BSP proteins and milk proteins [8]. With this dilution factor, an adequate amount of milk proteins was available to sequester all the BSP proteins present in the bovine, ram or stallion seminal plasma. For comparison purposes, we used the same ratio (1:20) to study the interaction of BSP proteins from ram and stallion with milk proteins. At this ratio, the amount of BSP distributed in various fractions after gel filtration chromatography was sufficient to detect by immunoblot. Since the concentration of BSP1 in boar SP is relatively low (~1 %), we used 1:1 ratio (35 mg boar SP proteins and 35 mg milk proteins) to study the interaction of BSP1 with milk proteins in this species. This dilution ratio permitted detection of BSP proteins in elution fractions following gel filtration chromatography. The protein content of the samples was determined by the modified Lowry procedure [33]. All gel filtration experiments using total seminal plasma proteins were performed in triplicate. Experiments performed using purified BSP proteins were done in duplicate.

### SDS-page and immunoblotting

Proteins were precipitated with trichloroacetic acid (TCA, 15 % (w/v) final concentration), reduced, denatured, and separated by electrophoresis on 15 % polyacrylamide gels. Analyses were performed by immunoblot or by Coomassie Blue R-250 staining. For immunoblots, following the transfer of proteins to PVDF membrane, membranes were blocked with PBS containing 1 % bovine serum albumin (BSA) and 0.02 % Tween-20 and then probed with affinity-purified polyclonal antibodies directed against a C-terminal 15-mer of the boar BSP1 (as described in [8]), stallion BSP proteins (antibodies isolated from the third boost at 1:1000 dilution) or ram BSP proteins (antibodies isolated from the third boost at 1:3000 dilution). All washings and antibody dilutions were done in PBS containing 0.1 % BSA and 0.02 % Tween-20. For identification, some proteins were also extracted from polyacrylamide gel and analyzed by liquid chromatography-tandem mass spectrometry (LC-MS/MS) as described by Havlis *et al*. [34].

### LC-MS/MS data analysis

Peaks were generated using Mascot Daemon v.2.1.6 while protein identification was performed with the Mascot software package v.2.1.03 (Matrix Science, London, UK) [35]. MASCOT was set up to search the nr_20090402 database (selected for Mammalia—720673 entries as on January 14^th^, 2010). The search criteria were as follow: Tryptic digestion; Variable modifications include carbamidomethylation (Cys) and oxidation (Met) with a peptide mass tolerance of ± 15 ppm and a fragment mass tolerance of ± 0.6 Da. The maximum missed cleavage number was set at 2.

## Results

### Generation of polyclonal antibodies

**Fig. 1:**
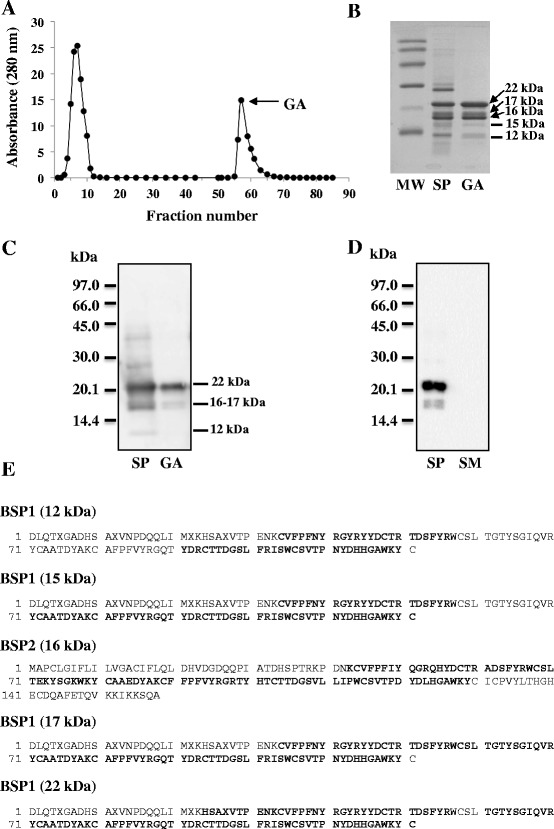
Isolation and identification of stallion BSP proteins. **a** Chromatogram of stallion SP proteins (150 mg) on gelatin-agarose column. The gelatin-adsorbed (GA) proteins (tubes 55–75) were pooled, desalted and freeze-dried. **b** SDS-PAGE pattern of the stallion SP proteins (15 μg) and of the GA fraction (10 μg). **c** Immunoblot analysis of stallion BSP proteins using the polyclonal antibodies directed against stallion BSP proteins. SP proteins (2 μg) and GA proteins (200 ng). **d** Specificity of the polyclonal antibodies directed against stallion BSP proteins. SP, stallion seminal plasma proteins (300 ng) and SM, skimmed milk proteins (10 μg). **e** LC-MS/MS analysis of the GA proteins. Bold letters represent residues identified by LC-MS/MS

In order to study the interaction of BSP proteins with milk components, polyclonal antibodies against BSP proteins from stallion and ram were raised. For polyclonal antibodies against stallion BSP proteins, proteins were purified from SP by affinity chromatography using their ability to bind strongly to gelatin. As seen on Fig. 1a, a large fraction of stallion SP proteins bound specifically to the gelatin-agarose column. We previously showed that stallion seminal plasma contains a group of BSP proteins with molecular mass of 12 kDa, 15–18 kDa, and 22–24 kDa [18]. Analysis of the gelatin-agarose adsorbed proteins by SDS-PAGE (Fig. 1b) revealed the presence of proteins with similar molecular weights. To confirm the identity of the proteins, each band was cut from the acrylamide gel and analyzed by LC-MS/MS spectrometry. The analysis showed that all the proteins recovered belong to BSP protein family. The bands corresponding to 22–24 kDa (generally detected as one broad band in immunoblots), 17 kDa, 15 kDa and 12 kDa were identified as BSP1, whereas that at 16 kDa was identified as BSP2 (Fig. 1e). Immunoblots were performed to test the specificity and sensitivity of the polyclonal antibodies raised against gelatin-adsorbed proteins. Two glycoforms of stallion BSP1 (22–24 kDa, 17 kDa), and stallion BSP2 (16 kDa) were detected (Fig. 1c). However, the antibodies did not detect the 15 kDa and 12 kDa stallion BSP proteins, probably due to their low concentration in SP [18]. As a control, it was validated that none of the milk proteins cross-reacted with antibodies (Fig. 1d).

**Fig. 2:**
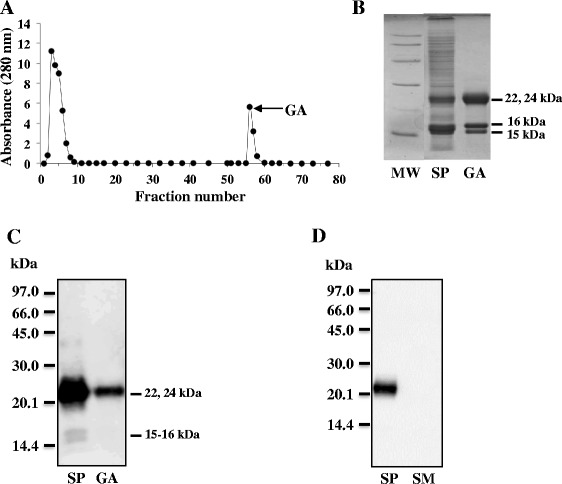
Isolation and identification of ram BSP proteins. **a** Chromatogram of ram SP proteins (150 mg) on gelatin-agarose column. The gelatin-adsorbed (GA) proteins (tubes 55–61) were pooled, desalted and freeze-dried. **b** SDS-PAGE of ram SP proteins (20 μg) and of GA fraction (15 μg). **c** Immunoblot analysis of ram BSP proteins using the polyclonal antibodies directed against ram BSP proteins. Ram SP proteins (2 μg) and GA proteins (200 ng). **d** Specificity of the polyclonal antibodies directed against ram BSP proteins. SP, ram seminal plasma proteins (300 ng) and SM, skimmed milk proteins (10 μg)

The same approach was used to produce polyclonal antibodies against ram BSP proteins. Purification of ram BSP proteins has already been described [20]. As previously reported, a large fraction of ram SP proteins bound specifically to the gelatin-agarose column (Fig. 2a). Gelatin-agarose bound proteins were separated by SDS-PAGE and bands of 15–16 kDa and 22–24 kDa, previously shown to be BSP proteins, were observed (Fig. 2b). Antibodies raised against the gelatin-binding proteins specifically recognized the 15–16 kDa and 22–24 kDa BSP proteins (Fig. 2c). They did not recognize any of the milk proteins (Fig. 2d).

### Milk proteins on Sepharose CL-4B

**Fig. 3:**
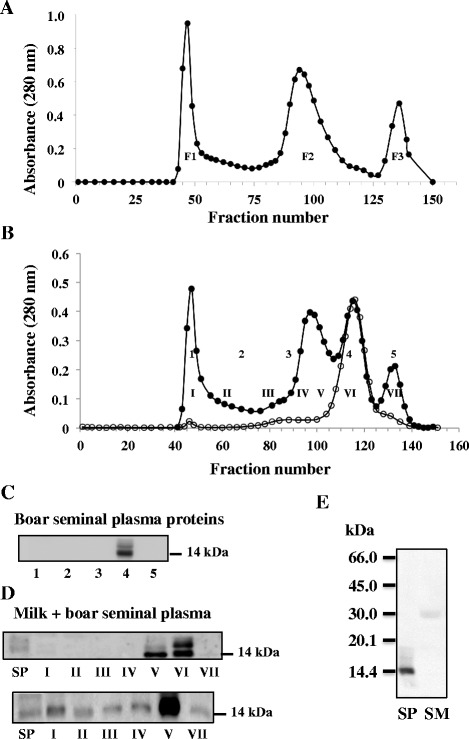
Gel filtration analysis of milk proteins and interaction between boar BSP1 and milk proteins. **a** Gel filtration chromatogram of 3 ml of heated skimmed milk alone. **b** Gel filtration chromatogram of 35 mg of boar SP proteins alone (open circles) or after incubation with skimmed milk (solid circles). Following the elution, tubes were pooled to provide five fractions for boar SP proteins alone (1, tubes 42–51; 2, tubes 52–78; 3, tubes 79–103; 4, tubes 104–128 and 5, tubes 129–139) or seven fractions for SP incubated with milk (I, tubes 42–54; II, tubes 55–71; III, tubes 72–88; IV, tubes 89–94; V, tubes 95–106; VI, tubes 107–124 and VII, tubes 125–138). **c** Immunoblot analysis of pooled fractions from the elution of boar SP proteins alone. Aliquots equivalent to 0.3 % of each fraction were precipitated and used for immunoblots. **d** Immunoblot analysis of pooled fractions from the elution of boar SP proteins incubated with skimmed milk. Proteins were precipitated and used for immunoblots (top panel: 0.015 % of fraction VI and 0.03 % of other fractions; bottom panel: 0.3 % of fractions). E) Immunoblot analysis of milk proteins with boar BSP1 polyclonal antibodies. Boar SP proteins (10 μg) and milk proteins (20 μg) were separated by SDS-PAGE, transferred to PVDF and probed with polyclonal antibodies against boar BSP1. The experiments were carried out in triplicates and the results of one typical experiment are shown

When chromatographed on a Sepharose CL-4B column, heated skimmed milk separates into three peaks (Fig. 3a). As previously described, the first peak (milk F1; tubes 40–60) contains mainly α-lactalbumin, β-lactoglobulin, κ-casein and some high-molecular-weight corresponding to albumin, lactoferrin, and immunoglobulins; the second peak (milk F2; tubes 80–110) contains mainly caseins (α- and β-caseins); and the third peak (milk F3; tubes 130–150) contains small-molecular-weight components such as amino acids, salts, sugars and vitamins [36].

To study interactions between SP proteins and these different milk fractions, SP alone or following incubation with milk was passed on a Sepharose CL-4B column and protein patterns were compared. Following elution, tubes collected from the columns were pooled. For SP alone tubes were pooled into five fractions (1–5): fractions 1, 3 and 5 corresponding to where milk F1, F2 and F3 were found respectively. Tubes collected for SP incubated with milk were pooled into seven fractions (I-VII). For those experiments, fractions I-II correspond to milk F1, fractions IV-V correspond to milk F2, while fraction VII correspond to milk F3 (Table 1). Equivalent proportions of the pooled fractions were precipitated and analyzed by immunoblot.

**Table 1:** Corresponding pooled fractions eluted from Sepharose CL-4B column (Fig. **3**a)

Milk fraction	Composition	Corresponding fractions SP alone	Corresponding fractions SP + Milk
Fraction 1 (tubes 40–60)	α-lactalbumin	1	I - II
	β-lactoglobulin		
	κ-casein		
Fraction 2 (tubes 80–110)	α-caseins	3	IV - V
	β-caseins		
Fraction 3 (Tubes 130–150)	amino acids	4	VII
	salts		
	sugars		
	vitamins		

### Interaction of boar SP proteins with milk proteins

Boar SP contains one BSP homolog named boar BSP1 (previously known as pB1) [6, 19]. Boar SP chromatographed on a Sepharose CL-4B column, gave rise to one large peak (Fig. 3b, open circles). Pooled fractions were subjected to immunoblot and boar BSP1 was detected in fraction 4 (Fig. 3c). Antibodies were specific for BSP1, as they did not cross-react with milk proteins or any other proteins found in boar SP (Fig. 3e).

Following incubation of boar SP with milk proteins at a 1:1 protein ratio, the protein mixture eluted from the Sepharose CL-4B column in four major peaks (Fig. 3b; solid circles). As observed in Fig. 3d (top panel), BSP1 was found in majority in fractions V and VI. Small amounts of boar BSP1 were also present in fractions I-IV (Fig. 3d, bottom panel), suggesting a strong interaction with milk F2 and a weaker interaction with milk F1.

**Fig. 4:**
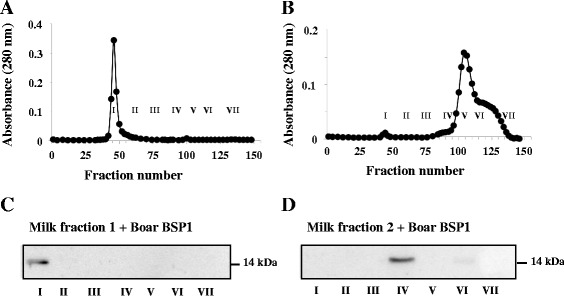
Gel filtration analysis of the interaction between pure boar BSP1 proteins and isolated milk fractions F1 and F2. **a**, **b** Chromatogram of purified boar BSP1 (100 μg) incubated with milk fraction F1 (2.9 mg) or milk fraction 2 (16.2 mg) respectively. Following the elution from both columns, tubes were pooled to provide seven fractions (I, tubes 41–47; II, tubes 48–67; III, tubes 68–87; IV, tubes 88–95; V, tubes 96–104; VI, tubes 105–113 and VII, tubes 114–146). **c**, **d** Immunoblot analysis of chromatographic fractions. Aliquots equivalent to 20 % of the pooled fractions were precipitated for the analysis. Duplicates were carried out and the results of one typical experiment are shown

To confirm this interaction, milk fractions F1 and F2 were separated, incubated individually with pure boar BSP1 and chromatographed on a Sepharose CL-4B column (Fig. 4a-b). When incubated with milk F1, BSP1 was found solely in fraction I, and when incubated with milk F2 alone, it was found only in fraction IV (Fig. 4c-d). This clearly demonstrates the interaction of boar BSP1 with both milk fractions.

### Interaction of stallion SP proteins with milk proteins

**Fig. 5:**
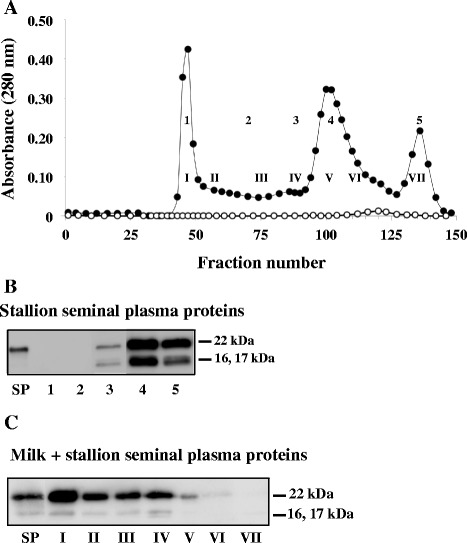
Gel filtration analysis of stallion SP proteins and interaction between stallion BSP proteins and milk proteins. **a** Gel filtration chromatogram of 1.75 mg of stallion SP proteins alone (open circles) or after incubation with skimmed milk (solid circles). **b** Immunoblot analysis of fractions from the elution of stallion SP proteins alone. Following the elution, aliquots equivalent to 6 % of tubes from five fractions (1, tubes 43–51; 2, tubes 52–74; 3, tubes 75–100; 4, tubes 101–126 and 5, tubes 127–141). **c** Immunoblot analysis of pooled fractions from the elution of stallion SP proteins incubated with skimmed milk. SP, stallion seminal plasma proteins (300 ng). Following elution, fractions were pooled in seven fractions (I, tubes 43–51; II, tubes 52–70; III, tubes 71–90; IV, tubes 91–100; V, tubes 101–110; VI, tubes 111–120 and VII, tubes 121–148). Aliquots equivalent to 0.3 % of each fraction were precipitated. The experiments were carried out in triplicates and the results of one typical experiment are shown

Similar experiments were conducted using Stallion SP. Seminal plasma alone eluted from the Sepharose CL-4B column as a small peak at the end of the elution pattern (Fig. 5a; open circles). Equivalent proportions of pooled fractions (1–5) were analyzed by immunoblot using polyclonal antibodies directed against stallion BSP proteins (Fig. 5b). Stallion BSP1 (22 kDa and 17 kDa) and BSP2 (16 kDa) were detected mainly in fractions 4 and 5. A faint amount of proteins was also detected in fraction 3.

To test the interaction with milk, stallion SP proteins: milk proteins ratio of 1:20 was used. The elution pattern of this mixture (Fig. 5a, solid circle) showed three major peaks corresponding to the milk proteins elution profile. The stallion BSP1 of 22 kDa was found in fractions I-V and stallion BSP2 of 16 kDa was detected in fractions I-IV (Fig. 5b). No proteins were detected in fractions VI and VII where stallion BSP proteins chromatographed alone were detected. These results indicate that the stallion BSP proteins interact strongly with whey protein aggregates (milk F1) and casein micelles (milk F2) although the interaction with milk F1 seems stronger.

### Interaction of ram SP proteins with milk proteins

**Fig. 6:**
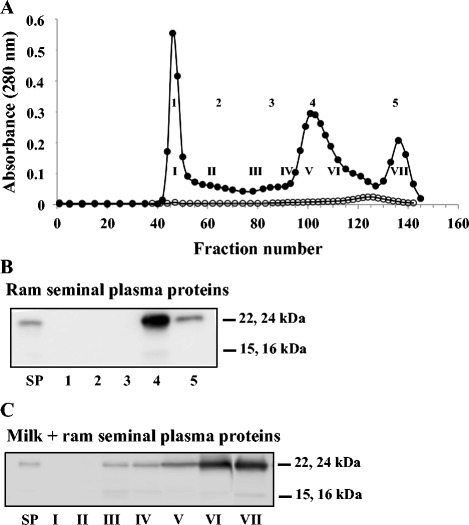
Gel filtration analysis of ram SP proteins and interaction between ram BSP proteins and milk proteins. **a** Gel filtration chromatogram of 1.75 mg of ram SP proteins alone (open circles) or after incubation with skimmed milk (solid circles). Following the elution, tubes were pooled to provide five fractions for ram SP proteins alone (1, tubes 40–50; 2, tubes 51–80; 3, tubes 81–100; 4, tubes 101–130 and 5, tubes 131–141) or seven fractions for SP incubated with milk (I, tubes 40–50; II, tubes 51–70; III, tubes 71–90; IV, tubes 91–100; V, tubes 101–110; VI, tubes 110–120 and VII, tubes 121–142). Aliquots equivalent to 3 % of each fraction were precipitated and used for immunoblots. **b** Immunoblot analysis of pooled fractions from the elution of ram SP proteins alone. SP, ram seminal plasma proteins (300 ng). **c** Immunoblot analysis of pooled fractions from the elution of ram SP proteins incubated with skimmed milk. The experiments were carried out in triplicates and the results of one typical experiment are shown

When ram SP proteins were loaded into the Sepharose CL-4B column, a small protein peak was observed (Fig. 6a, open circles). Immunoblot of equivalent proportions of the pooled fractions was performed using polyclonal antibodies directed against ram BSP proteins. The polyclonal antibodies recognized mainly the 22–24 kDa ram BSP proteins, which were detected strongly in fraction 4 and weakly in fraction 5 (Fig. 6b). A very faint band corresponding to the 15–16 kDa ram BSP proteins was detected in fraction 4, possibly because of their very low concentration in ram SP and of the dilution factor associated with gel filtration.

The interaction between ram BSP proteins and milk proteins was studied using SP proteins with milk at a protein ratio of 1:20. The proteins eluted from the Sepharose CL-4B column as three major peaks (Fig. 6a, solid circles). Ram BSP of 15–16 and 22–24 kDa were not detected in fractions I-II but were detected in increasing amount from fraction III through VII (Fig. 6c), indicating an interaction between Ram BSP proteins and milk F2. However, majority of the 22–24 kDa proteins were found in fractions VI and VII, suggesting that the interaction with milk F2 is either weak or rapidly saturated.

**Fig. 7:**
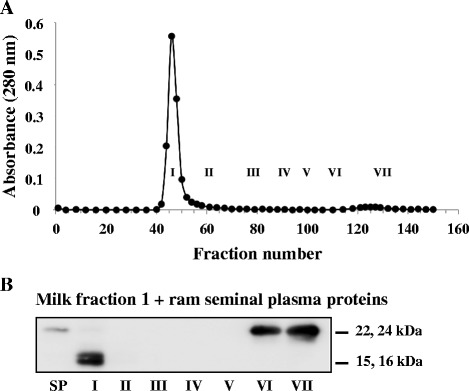
Gel filtration analysis of the interaction between gelatin-adsorbed ram BSP proteins and milk fraction F1. **a** Chromatogram of gelatin-adsorbed ram BSP proteins (500 μg) incubated with milk fraction F1 (4.8 mg). Tubes were pooled to provide seven fractions (I, tubes 42–52; II, tubes 53–70; III, tubes 71–90; IV, tubes 91–100; V, tubes 101–110; VI, tubes 111–120 and VII, tubes 121–150). **b** Immunoblot analysis of chromatographic fractions. SP: ram SP proteins (300 ng). Aliquots equivalent to 5 % of the fractions were precipitated. Duplicates were carried out and the results of one typical experiment are shown

No clear indication of the binding of ram BSP proteins with milk F1 was observed. To investigate it ram gelatin-bound SP proteins (containing BSP proteins only) were incubated with milk F1 alone and chromatographed. Elution pattern showed only one large peak corresponding to milk F1 (Fig. 7a). The analysis of proteins demonstrated that BSP of 15–16 kDa were present in fraction I, whereas BSP 22–24 kDa were only found in fractions VI-VII (Fig. 7b).

## Discussion

### Interaction of BSP homologs with the bovine milk proteins

Our prior studies revealed that extended contact of bull BSP proteins with sperm has a detrimental effect on sperm [37]. We also showed that through an association of these proteins with casein micelles and whey protein aggregates present in milk extender, the amount of BSP proteins bound to sperm surface decreases thereby preventing or minimizing cholesterol and phospholipid loss from the sperm membrane and protecting sperm during storage [13, 14]. The interaction between bovine BSPs and milk proteins was investigated in detail by gel filtration and by isothermal calorimetry (ITC). Both of these techniques confirmed that BSP proteins interact with milk proteins (whey proteins and casein micelles) and that this interaction displays low affinity and low capacity [14]. Similar observations were made recently in goat where BSP proteins were shown to interact with caseins and β-lactoglobulin. This interaction was shown to decrease the binding of BSP proteins to sperm (Menezes *et al*. manuscript in preparation). In the current study, we demonstrated that BSP homologs in boar, stallion and ram seminal plasma share the binding properties of the bovine BSP for the milk proteins. In all instances, a large amount of BSP proteins co-eluted with milk proteins during gel filtration chromatography. These studies broadened our novel concept that the interaction of extender components with seminal plasma proteins is the basis of sperm protection.

Even though, the association of BSP proteins with milk proteins appears to be a general feature, some distinctions could be observed between the different systems. In our previous study, the three bovine BSP proteins (BSP1, BSP3 and BSP5) were found to interact in a similar affinity with casein micelles (milk F2), but only BSP1 and BSP5 seemed to interact with whey protein aggregates (milk F1) [14]. In the current study, boar BSP1 was found to bind mainly to milk F2, suggesting a higher affinity of the protein for caseins than for whey proteins (milk F1). Stallion BSP1 (22 kDa and 17 kDa) and BSP2 (16 kDa) interacted strongly with both whey protein aggregates (milk F1) and casein micelles (milk F2). However, since more BSP proteins were co-eluted with milk F1, it is inferred that stallion BSP proteins have a higher affinity for whey protein aggregates than for caseins. Results also showed that the ram 15–16 kDa BSP proteins appear to favor binding to whey protein aggregates, whereas the 22–24 kDa BSP proteins bound preferentially, but with low capacity, to casein micelles.

These association differences could be due to structural differences among the BSP proteins. All the BSP proteins possess two fibronectin type 2 (Fn2) domains (C-terminal part) and a variable N-terminal part. However, some discrepancies regarding binding preferences of the different members of the BSP superfamily have been reported. BSP proteins are glycosylated to different extent and some like bovine BSP5 being highly glycosylated, while others such as bovine BSP3 are not glycosylated at all. Thus, variations in their sequence, structure and post-translational modifications, may account for some differences in their binding affinities for ligands. For example, goat BSP proteins of 20–22 kDa have a strong affinity for heparin whereas goat BSP proteins of 14–15 kDa display only a weak affinity for this ligand [16].

In the bovine species, we have demonstrated that, during storage, less BSP proteins are physically associated with sperm membranes when milk proteins are present in the extender [13]. The physical association of BSP proteins and milk proteins remains even after freeze-thaw cycles (unpublished data). Our studies with BSP proteins from five different species strongly suggest that the affinity of these proteins for milk proteins is a general phenomenon and that this association could lead to reduced binding of BSP proteins to sperm and contributes to the protection of sperm during storage.

### Mechanism of sperm protection by milk

The current study together with previous work [14] corroborate that BSP proteins in general interact with whey protein aggregates and/or casein micelles. Although, there are differences in binding affinity of BSP proteins for whey proteins and/or casein micelles, it is remarkable that all BSP proteins in the SP of bull, goat, boar, stallion and ram have affinity for the milk proteins. Bison BSP homologs, which share sequence identity and binding properties with the bovine proteins, are also likely to have affinity for the milk proteins [15, 16, 20]. In view of this and other prior studies [13, 14], we believe that, similarly to the mechanism proposed for egg yolk extenders in which LDL sequestrate BSP proteins and protect sperm during storage, milk extender could also protect sperm by sequestrating the BSP proteins [2, 37]. It is well established that when high concentrations of free BSPs are in contact with sperm they begin to extract lipids from the membranes [2]. This continuous exposure leads to an extensive loss of lipids from sperm membranes, which may be detrimental to sperm storage. Thus, the interaction of BSP proteins with whey proteins and casein micelles could decrease the amount of free BSP proteins, limit lipid loss from sperm membranes and hence protect sperm during storage.

We believe that the present findings should encourage studies assessing the protective impact of BSP protein sequestration by extender components for various species. Sperm preservation is unquestionably based on complex interactions and involves several parameters including the nature of the BSP proteins, their relative abundance, and the timeline of the preservation process (e.g., the moment of the addition of the extender) [38]. For example, several studies in rams have reported that the addition of seminal plasma to sperm before or after cold-shock could help repair/prevent cryoinjuries. This protective effect was partly attributed to ram BSP-15 kDa and BSP-22 kDa [39–45]. These experiments showing the positive effect of ram seminal plasma on sperm membranes have been inferred from short-term incubations of 30–60 min. Interestingly, results obtained in bovine suggest that BSP proteins are detrimental to sperm only when they are incubated for long periods and/or high concentrations. In fact, in bovine, no noticeable effect on phospholipid efflux from sperm membrane is observed in the presence of BSP1 and BSP3 before 1 h of incubation [46]. Similar conclusions can be deduced in stallions where seminal plasma was shown to have little effect (positive or negative) on sperm viability and motility if sperm were frozen immediately after processing, but was detrimental for cryosurvival if sperm were in contact with high levels of seminal plasma for long periods prior to freezing [47]. Therefore, it appears that the kinetics aspect of the sample preparation for preservation is critical, that incubation of sperm in the presence of seminal plasma could help to stabilize the plasma membrane for short periods, but that longer incubation or high concentrations of SP would have detrimental effect. This time aspect should be examined in various species and conditions in order to assess in detail the putative positive impact provided by the general reduction of available BSP protein content due to sequestration with milk proteins reported here.

On the other hand, the studies performed in ram also demonstrate that sperm from animals expressing higher concentrations of BSP proteins tend to resist better to cryopreservation and that ram BSP proteins tend to stabilize the sperm membranes better than bovine or equine BSP proteins [38, 45, 48]. Furthermore, although several studies prove that seminal plasma in bull, boar and stallion is detrimental for sperm, no such observation have been reported in ram [42, 47, 49]. Thus, it is possible that BSP proteins in ram are not as detrimental as the ones found in bull, boar or stallion. Concurrently, results in the actual study show that BSP in ram do bind to milk proteins, but seem to have a low binding capacity indicating that milk proteins possibly sequester part of the ram BSP proteins, but still leave high concentration free to stabilize and decapacitate sperm.

The concentration of BSP protein homologs in the SP varies from <1 % in boar to as high as 50 % in bovine semen [2]. In the semen processing industries, each ejaculate is diluted with the extender according to the sperm concentration. On the basis of the protective mechanism offered by milk proteins as proposed here, it could be advantageous, in order to improve the sperm protection by milk, to use a dilution ratio of the semen with the milk extender that is tailored for each species according to the BSP concentration in semen. In species with a high BSP protein concentration, a higher dilution ratio may provide more protection to sperm. The optimization of the dilution ratio should however permit some BSP proteins to interact with sperm because they are required for sperm capacitation (reviewed in [7]).

## Conclusion

In summary, we demonstrated that BSP proteins present in boar, ram and stallion SP have affinity for the milk proteins just like bovine and goat proteins. These results support our contention that the mechanism of sperm protection by milk could involve common features in all these species. The results presented in this paper may aid in improving the efficiency of the milk extenders and in developing novel extenders free of product of animal origin.
